# The role of microRNAs in calcific aortic valve disease

**DOI:** 10.3389/fcvm.2025.1637753

**Published:** 2026-01-09

**Authors:** Yi-Zhou Peng, Yang He, Yue-Jiao Yang, Liang Tang

**Affiliations:** Department of Cardiology, The Second Xiangya Hospital of Central South University, Changsha, Hunan, China

**Keywords:** aortic valve interstitial cells, calcific aortic valve disease, mechanism, microRNAs, osteogenic differentiation

## Abstract

Calcific aortic valve disease (CAVD) is a prevalent and progressive valvular disorder characterized by fibrocalcific remodeling and eventual valve stenosis. Despite its clinical burden, current treatment is limited to valve replacement at end-stage disease, with no approved medical therapy to slow or halt its progression. Increasing evidence suggests that microRNAs (miRNAs), small non-coding RNAs that post-transcriptionally regulate gene expression, are critically involved in the pathophysiology of CAVD. This review highlights the roles of key miRNAs in CAVD, focusing on their involvement in endothelial dysfunction, inflammation, extracellular matrix remodeling, and the osteogenic differentiation of valvular interstitial cells. Furthermore, we explore the intersection between CAVD and osteoporosis, two diseases that share overlapping pathophysiological mechanisms and miRNA regulators. Understanding miRNA-mediated pathways in CAVD may uncover novel biomarkers and therapeutic targets to delay disease progression and improve patient outcomes.

## 1 Introduction

Calcific aortic valve disease (CAVD) is the most common form of valvular heart disease, particularly in aging populations. The disease is characterized by the progressive accumulation of fibrocalcific material on the aortic valve leaflets, which leads to stenosis and left ventricular outflow obstruction (1). Over time, this results in pressure overload, compensatory left ventricular hypertrophy, and ultimately heart failure. While the prevalence of CAVD continues to rise, especially in industrialized nations, there are currently no effective pharmacological therapies to prevent or slow its progression (2). Surgical aortic valve replacement or transcatheter aortic valve implantation remains the only definitive treatment for severe aortic stenosis (3).

In 2017, the global prevalence of degenerative mitral valve disease was estimated at 12.6 million cases. During the same year, calcific aortic valve disease accounted for approximately 102,700 deaths worldwide, whereas degenerative mitral valve disease was responsible for an estimated 35,700 deaths. Between 1990 and 2017, aging and population growth contributed to a 112% increase in mortality attributable to nonrheumatic valvular diseases (4).

Historically considered a passive, age-related degenerative condition, CAVD is now recognized as an active disease process driven by valvular endothelial cells (VECs) dysfunction, chronic inflammation, lipid infiltration, extracellular matrix (ECM) remodeling, and osteogenic differentiation of aortic valvular interstitial cells (VICs). Among the various regulatory molecules involved in this complex pathophysiology, miRNAs have emerged as critical post-transcriptional regulators that modulate a wide range of biological processes. MiRNAs are approximately 22 nucleotides in length and act by binding to complementary sequences in the 3′ untranslated region (3′ UTR) of target mRNAs, leading to either mRNA degradation or inhibition of translation (5, 6).

In cardiovascular diseases, miRNAs have been shown to influence cardiac development, angiogenesis, fibrosis, and pathological remodeling (7). While previous reviews have summarized the roles of miRNAs in CAVD (8–10), emerging evidence continues to uncover how additional aberrant miRNA expression profiles critically regulate disease mechanisms. This review provides a systematic and mechanism-driven synthesis, emphasizing the distinct functions of specific miRNAs—such as pro-osteogenic miR-101-3p, anti-calcific miR-204, and hemodynamics-sensitive miR-181b—in driving endothelial dysfunction, inflammatory activation, extracellular matrix remodeling, and osteogenic differentiation of valvular interstitial cells. A major conceptual advance presented here is the explicit delineation of the mechanistic overlap between CAVD and osteoporosis, demonstrating how shared miRNA regulators simultaneously promote ectopic valve calcification and systemic bone loss. By integrating these insights, our work not only refines the understanding of miRNA functions in CAVD but also proposes potential novel therapeutic strategies that exploit the dual role of miRNAs in cardiovascular and skeletal pathologies.

## 2 Pathophysiology of calcific aortic valve disease

CAVD is now understood as a chronic, actively regulated disease that involves multiple stages: initiation, propagation, and end-stage calcification (2). The initiation phase is marked by VECs dysfunction, particularly on the aortic side of the valve, where turbulent flow, mechanical stress, and oxidative stress lead to increased permeability and lipid infiltration (11–13). Modified lipids, such as oxidized low-density lipoprotein, initiate an inflammatory response within the valvular endothelium, characterized by the recruitment and activation of macrophages, T lymphocytes, and mast cells (12). At this stage, punctate microcalcification typically colocalizing with regions of lipid deposition.

In contrast to the initiation phase, the propagation phase is predominantly characterized by progressive fibrosis and calcification, rather than by ongoing inflammation and lipid accumulation. Fibrosis promotes the deposition of a collagen-rich extracellular matrix, which serves as a structural scaffold that facilitates the subsequent development of progressive calcification. This fibrotic process within the valve may be partially driven by diminished nitric oxide bioavailability secondary to endothelial injury (14). In addition, upregulation of angiotensin-converting enzyme in calcific aortic valve disease, where low-density lipoprotein enhances the conversion of angiotensin I to angiotensin II, thereby promoting pro-fibrotic signaling via the angiotensin II type 1 receptor (15). The phenotypic transition of VICs into an osteoblast-like lineage is considered a pivotal mechanism driving the acceleration of valvular calcification. VICs become activated and acquire myofibroblastic and osteoblast-like phenotypes under pathological conditions (2). These activated VICs express bone-associated proteins such as Runt-related transcription factor 2 (Runx2), osteocalcin, alkaline phosphatase (ALP), and osteopontin, leading to hydroxyapatite deposition and progressive valve stiffening. Multiple signaling pathways, including Notch, bone morphogenetic protein (BMP), Wnt/β-catenin, and transforming growth factor-beta (TGF-β) are implicated in regulating this phenotypic switch and interact with miRNAs to modulate disease progression (16–18).

## 3 MicroRNAs in CAVD: molecular insights

This section provides a detailed analysis of key miRNAs implicated in CAVD, emphasizing their regulatory roles in VIC osteogenesis, inflammation, and ECM remodeling.

### 3.1 Pro-Calcific miRNAs as mediators of osteogenic activation

#### 3.1.1 miR-101-3p

miRNA-101 is a highly conserved non-coding RNA (19). miR-101-3p, named for the cleavage of the 3′ arm of pre-miR-101 by the Dicer enzyme, is a predominant mature form of miR-101 (20). While miRNA-101 has historically been extensively studied in cancer research (21), recent evidence suggests it also plays a regulatory role in cardiovascular biology, including the inhibition of endothelial cell proliferation and myocardial fibrosis (22).

A study by Chen et al. (23). investigated the role and underlying mechanisms of miR-101-3p in human aortic valve interstitial cells (HAVICs) calcification. The researchers found that miR-101-3p expression was significantly elevated in calcified aortic valves compared to normal controls. In cultured HAVICs, overexpression of miR-101-3p promoted calcification and activated the osteogenic signaling pathway. Conversely, inhibition of miR-101-3p suppressed osteogenic differentiation and calcification, even in osteogenic induction media. Mechanistically, miR-101-3p directly downregulated the expression of cadherin-11 (CDH11) and SRY-box transcription factor 9 (SOX9), both of which are essential for chondrogenesis and osteogenesis. Suppression of miR-101-3p restored CDH11 and SOX9 levels and effectively blocked the osteogenic process in VICs.

#### 3.1.2 miR-22

miR-22 is encoded by MIR-22HG (the miR-22 host gene), which is located within an exon of a long non-coding RNA. MIR-22HG resides in the intergenic region between two protein-coding genes, TLCD2 (TLC domain containing 2) and WDR81 (WD repeat domain 81) (24). Deep sequencing analyses have shown that miR-22 is among the most abundant microRNAs in the heart (25). Previous studies demonstrated that miR-22 agomiR (a miRNA mimic) inhibits neointima formation in injured mouse femoral arteries and promotes the phenotypic transition of vascular smooth muscle cells from a proliferative, synthetic state to a contractile phenotype under pathological conditions (26).

Yang et al. (27) found that miR-22 expression is significantly elevated in calcified aortic valves from patients with CAVD, and its expression is positively correlated with osteogenic markers such as osteopontin (OPN) and Runx2 in VICs. *In vitro*, overexpression of miR-22 accelerated the osteogenic differentiation of VICs, as evidenced by increased calcium deposition, elevated alkaline phosphatase activity, and upregulation of osteoblastic markers. Conversely, inhibition of miR-22 suppressed these effects, suggesting that miR-22 promotes VIC osteogenesis. The authors also identified calcium-binding protein 39 (CAB39) as a direct target of miR-22. Overexpression of miR-22 reduced CAB39 expression, thereby inhibiting the CAB39–LKB1–STRAD complex and disrupting the adenosine monophosphate-activated protein kinase (AMPK)–mammalian target of rapamycin (mTOR) signaling pathway, ultimately promoting VIC calcification. These findings suggest that miR-22 plays a crucial role in promoting osteogenic differentiation in CAVD.

### 3.2 Anti-calcific miRNAs as inhibitors of osteogenic signaling

#### 3.2.1 miR-638

Human miR-638 is located on chromosome 19p13.2 (28). Previous studies have shown that miR-638 plays a critical role in the pathogenesis of several tumors (29–31), emphysematous lung destruction (32), and diabetic nephropathy (33). More recently, a number of studies have reported that reduced miR-638 expression is associated with carotid atherosclerosis and high-grade stenosis (34).

Jiao et al. (35) investigated the function of miR-638 in the calcification of AVICs and found that miR-638 expression was significantly elevated in calcified aortic valves compared to non-calcified control. *In vitro* experiments demonstrated that overexpression of miR-638 suppressed osteogenic differentiation of AVICs, as indicated by reduced ALP activity and lower expression of osteogenic markers such as ALP and integrin-binding sialoprotein (IBSP). In contrast, silencing miR-638 enhanced osteogenic differentiation. Dual-luciferase reporter assays confirmed that miR-638 binds to the 3′ untranslated region (UTR) of specificity protein 7 (Sp7), key transcription factor involved in osteogenesis. Moreover, Sp7 silencing mimicked the effects of miR-638 overexpression by inhibiting osteogenic differentiation. The inhibitory effect of miR-638 on osteogenesis was partially reversed by Sp7 knockdown, collectively indicating that miR-638 regulates osteogenic differentiation through suppression of Sp7.

#### 3.2.2 miR-214

The microRNA miR-214 is located at chromosomal region 1q24.3, within intron 14 of the Dynamin-3 (DNM3) gene (36). miR-214 plays a pivotal role in the pathogenesis of various diseases, including multiple cancers (37, 38), pulmonary arterial hypertension (39), and osteonecrosis (40). Notably, exosomes derived from engineered human umbilical mesenchymal stem cells expressing miR-214 have demonstrated cardioprotective effects by attenuating myocardial fibrosis (41).

Li et al. (42) investigated the role of miR-214 in the development of CAVD. They observed a significant reduction in miR-214 expression in aortic valve leaflets from CAVD patients, accompanied by increased expression of osteogenic markers such as Runx2, Sp7, activating transcription factor 4, and OPN. *In vitro*, miR-214 overexpression suppressed osteogenic differentiation of VICs, whereas silencing miR-214 enhanced this process. Dual-luciferase reporter assays and rescue experiments confirmed ATF4 and Sp7 as direct targets of miR-214. *In vivo*, miR-214 knockout rats exhibited exacerbated aortic valve calcification and elevated mean transvalvular velocity. Collectively, these findings underscore the essential role of miR-214 in inhibiting osteogenic differentiation and protecting against valve calcification.

#### 3.2.3 miR-204

miR-204 is encoded within intron 9 of the TRPM3 gene and gives rise to a 110-base pair (bp) pre-microRNA-204 stem-loop structure (43). Its expression exhibits a highly tissue-specific pattern. Analysis of human tissues has shown that miR-204-5p is particularly enriched in neural tissues, including the brain and spinal cord (44). Detectable levels of miR-204-5p have also been observed in arterial tissue.

Song et al. (45) reported that miR-204 expression was significantly reduced in calcified human aortic valves and in VICs isolated from these valves, compared to normal controls. Functional studies demonstrated that transfection with a miR-204 mimic suppressed ALP expression and calcium deposition in diseased VICs, whereas inhibition of miR-204 enhanced the expression of osteogenic markers such as Runx2, Osterix (Osx), and ALP in normal VICs. Further experiments showed that miR-204 directly targets Runx2 and Osx, key transcription factors involved in osteogenesis, thereby blocking TGF-β1-induced osteogenic reprogramming. *Ex vivo* studies using mouse aortic valves confirmed that antagonism of miR-204 upregulated Runx2 and Osx, accelerating calcium deposition, while silencing these targets attenuated calcification. Collectively, these findings identify miR-204 as a critical suppressor of valvular osteogenic activity.

#### 3.2.4 miR-449c-5p

MicroRNA-449c-5p is a member of the miR-34/449 family, which comprises six homologous miRNAs: miR-34a/b/c and miR-449a/b/c (46). Previous studies have demonstrated that members of this family play critical roles in cancer (47, 48), brain development (49), and spermatogenesis (50). Additionally, miR-449a-5p has been identified as a negative regulator of cardiomyocyte proliferation (51).

Xu et al. (52) reported that miR-449c-5p expression is significantly downregulated in calcified aortic valves compared to non-calcified valves. *In vitro* experiments demonstrated that overexpression of miR-449c-5p inhibits osteogenic differentiation of VICs, whereas its downregulation enhances this process. The study further identified mothers against decapentaplegic homolog 4 (Smad4), a key mediator of the TGF-β/Smad signaling pathway, as a direct target of miR-449c-5p. Target prediction analyses and dual-luciferase reporter assays confirmed miR-449c-5p binding to the 3′ untranslated region (3′UTR) of Smad4. Moreover, knockdown of Smad4 in VICs inhibited osteogenic differentiation, mimicking the effects of miR-449c-5p overexpression. *In vivo*, overexpression of miR-449c-5p significantly reduced aortic valve calcification in a mouse model. These findings suggest that miR-449c-5p may serve as a promising therapeutic target for attenuating valve calcification CAVD.

#### 3.2.5 miR-138-5p

The miR-138-5p sequence is highly conserved among animals. Two independent genes located on chromosomes 3 and 16 encode miR-138 (53). Numerous studies have documented the role of miR-138 in various cancers, diabetic nephropathy (54), and pulmonary fibrosis (55).

Yan et al. (56) reported that miR-138-5p expression is significantly downregulated in patients with CAVD and during the osteogenic differentiation of VICs. Overexpression of miR-138-5p reduced ALP activity and decreased the expression of osteogenic marker proteins such as OPN and ALP, ultimately inhibiting osteogenic differentiation and calcium deposition in VICs. Conversely, silencing miR-138-5p enhanced osteogenesis. Further analysis identified RUNX2, a key osteogenic transcription factor, as a direct target of miR-138-5p. Dual-luciferase reporter assays confirmed the interaction between miR-138-5p and the 3′ untranslated region (UTR) of RUNX2. A negative correlation between miR-138-5p and RUNX2 expression was also observed in CAVD tissue samples. Moreover, overexpression of RUNX2 reversed the inhibitory effects of miR-138-5p on osteogenic differentiation, supporting a regulator *y* axis involving miR-138-5p and RUNX2. Additionally, the study revealed that the Wnt/β-catenin signaling pathway is implicated in miR-138-5p–mediated regulation. Overexpression of miR-138-5p suppressed the expression of Wnt5a and β-catenin proteins, and this effect was reversed by RUNX2 overexpression. These findings suggest that miR-138-5p regulates osteogenic differentiation through the RUNX2–Wnt/β-catenin axis.

#### 3.2.6 miR-195

miR-195 is a member of the miR-15 family and is located on chromosome 13q14 (57, 58). Previous research on miR-195 has primarily focused on its role in various cancers (59–61), diabetes (62), and non-alcoholic fatty liver disease (NAFLD) (63). In the cardiovascular field, recent studies have shown that homocysteine-mediated miR-195-3p promotes the progression of atherosclerosis by targeting interleukin-31 (IL-31) (63).

Yang et al. (64) reported that miR-195 is significantly downregulated in CAVD tissues, whereas the expression of von Willebrand factor (VWF)—a protein associated with endothelial dysfunction—is markedly increased. Their subsequent experiments demonstrated that miR-195 directly targets VWF and negatively regulates its expression, thereby inhibiting the p38 MAPK signaling pathway and alleviating aortic valve calcification. Upregulation of miR-195 in VICs reduce ALP activity and calcium deposition *in vitro*, suggesting that miR-195 suppresses osteogenic differentiation. Similarly, silencing VWF also decreased AVIC calcification, further supporting the role of the miR-195/VWF axis as a key regulator in CAVD. Finally, Western blot analysis confirmed that both miR-195 overexpression and VWF knockdown reduced phosphorylation of p38 MAPK. These findings collectively indicate that miR-195 exerts a protective effect against CAVD by inhibiting VWF and suppressing the p38 MAPK signaling pathway.

### 3.3 miRNAs as regulators of the pathogenic microenvironment

#### 3.3.1 miR-29

The microRNA-29 (miR-29) family comprises three mature members: miR-29a, miR-29b, and miR-29c. Among them, miR-29b (5ʹ-CUGGUUUCACAUGGGUGGCUUAG-3ʹ) is a small non-coding RNA of 22 nucleotides in length (65). The miR-29 family plays crucial roles in various cellular processes, including extracellular matrix homeostasis, collagen synthesis, glucose metabolism, and fibrosis. It has also been proposed as a potential predictive biomarker for the early diagnosis of malignant tumors and other pathological condition (66, 67).

Fang et al. (68) reported that NOD-like receptor family pyrin domain containing 3 (NLRP3) is a key component of the inflammasome and is upregulated in patients with CAVD, whereas the expression of suppressor of cytokine signaling 1 (SOCS1) a negative regulator of NLRP3—is significantly reduced. miR-29b leading to activation of the NLRP3 inflammasome and enhancing inflammatory responses by suppressing the signal transducer and activator of transcription 3 (STAT3)/SOCS1 signaling pathway, which contribute to osteoblastic differentiation in VICs.

Wang et al. (69) investigated the role of miR-29a-5p in parathyroid hormone (PTH)-induced valvular calcification in chronic kidney disease (CKD). They observed a significant reduction in miR-29a-5p expression in aortic valves from CKD rats, accompanied by decreased expression of the endothelial marker CD31 and increased expression of mesenchymal markers such as α-SMA, FSP1, CD44, and CD10. *In vitro*, PTH stimulation suppressed miR-29a-5p, leading to Endothelial-to-mesenchymal transition (EndMT) in endothelial cells, whereas overexpression of miR-29a-5p, GSAP knockdown, or γ-secretase inhibition (DAPT) abrogated this process. Dual-luciferase reporter assays and rescue experiments confirmed gamma-secretase activating protein (GSAP) as a direct target of miR-29a-5p, linking its repression to Notch1 pathway activation through increased Notch intracellular domain (NICD) and hairy and enhancer of split-1 (Hes1). *In vivo*, delivery of adeno-associated virus (AAV)–miR-29a-5p or treatment with DAPT significantly blunted EndMT, restored CD31 expression, reduced NICD/Hes1 levels, and attenuated calcium–phosphate deposition in CKD rat aortic valves. Collectively, these findings underscore the essential role of miR-29a-5p in restraining PTH-induced EndMT via GSAP/Notch1 signaling and protecting against valvular calcification.

#### 3.3.2 miR-181b

The miRNA-181 family comprises four members: miR-181a, miR-181b, miR-181c, and miR-181d (70). Among them, miR-181b has been implicated in various pathological processes, including diabetic nephropathy (71), cognitive dysfunction (72), and the proliferation and differentiation of chondrocytes (73). More recently, in the context of cardiovascular disease, miR-181b has been shown to TIMP3, thereby contributing to the development of atherosclerosis (74). It is also involved in endothelial-to-mesenchymal transition associated with atrial fibrillation (75).

Heath et al. (76) reported that miR-181b is upregulated in the fibrosa layer of the aortic valve under disturbed flow conditions, such as oscillatory shear stress (OS). Using luciferase reporter assays, they demonstrated that miR-181b directly binds to the 3′ untranslated region (UTR) of TIMP3, suppressing its expression. As TIMP3 is an endogenous inhibitor of matrix metalloproteinases (MMPs), its downregulation results in elevated MMP activity. In VECs exposed to OS, overexpression of miR-181b reduced TIMP3 levels, increased MMP activity, and promoted extracellular matrix remodeling. Conversely, inhibition of miR-181b reversed these effects, highlighting its critical role in regulating matrix dynamics. Collectively, these findings suggest that miR-181b modulates the TIMP3/MMP axis and plays a pivotal role in the pathological remodeling of the aortic valve under disturbed flow conditions.

### 3.4 Circulating microRNAs as diagnostic and prognostic biomarkers

Although tissue-level studies have elucidated the central role of miRNAs in the pathogenesis of CAVD, their potential as non-invasive “liquid biopsy” tools are particularly critical for clinical translation. Due to their high stability in the circulatory system and ease of detection, circulating miRNAs have emerged as novel biomarkers for the early diagnosis, severity assessment, and prognosis of CAVD (7, 10).

Multiple studies have demonstrated a significant correlation between circulating miRNA expression profiles and valvular pathological changes. miR-21 is currently one of the most extensively studied miRNAs; multiple independent studies have consistently found significantly elevated plasma miR-21 levels in patients with CAVD, which are closely associated with the degree of valvular interstitial fibrosis and left ventricular remodeling (7, 77). In contrast, other miRNAs exhibit a downregulation trend in the disease state. For instance, Galeone et al. (78) recently performed high-throughput analysis of calcified valve tissue and matched serum samples, finding that miR-17-5p and miR-29a-3p were significantly downregulated in both tissue and serum. This suggests that alterations in circulating miRNAs can mirror local valvular pathological processes, particularly extracellular matrix dysregulation and the activation of osteogenic differentiation signals.

Beyond diagnostic value, specific circulating miRNAs also demonstrate significant prognostic potential. A study by Røsjø et al. (79) indicated that elevated serum miR-210 levels were significantly associated with increased mortality in patients with moderate to severe aortic stenosis. Furthermore, circulating miRNAs have shown promise in predicting prognosis following aortic valve replacement (AVR) or transcatheter aortic valve implantation (TAVI). For example, patients with higher preoperative plasma miR-133a levels tended to exhibit greater left ventricular (LV) mass regression postoperatively, suggesting this biomarker may help identify patients who would derive maximal myocardial recovery benefit from surgery (80). Similarly, Eyileten et al. (81) found that baseline miR-223 levels were an independent predictor of major adverse cardio-cerebrovascular events following TAVI.

However, despite promising prospects, current research on miRNA biomarkers still faces numerous limitations that hinder their translation into clinical practice. Firstly, there is a lack of consistency across different studies. A systematic review by Adewuyi et al. (77) pointed out that among hundreds of differentially expressed miRNAs reported in the literature, only a few (such as miR-21 and miR-133a) have been validated in more than two studies. Secondly, existing studies are mostly single-center, small-sample cross-sectional studies, and patients often have comorbidities such as coronary artery disease, diabetes, or renal insufficiency, all of which may interfere with circulating miRNA expression profiles (7, 77). Additionally, the lack of standardization in sample processing (serum vs. plasma), extraction methods, and normalization controls contributes to the heterogeneity of study results. Therefore, future research requires validation of the specificity and sensitivity of candidate miRNAs in large-scale, multi-center prospective cohorts and the establishment of standardized detection protocols (7, 78).

## 4 Shared miRNA regulatory networks in CAVD and osteoporosis

**Figure 1:**
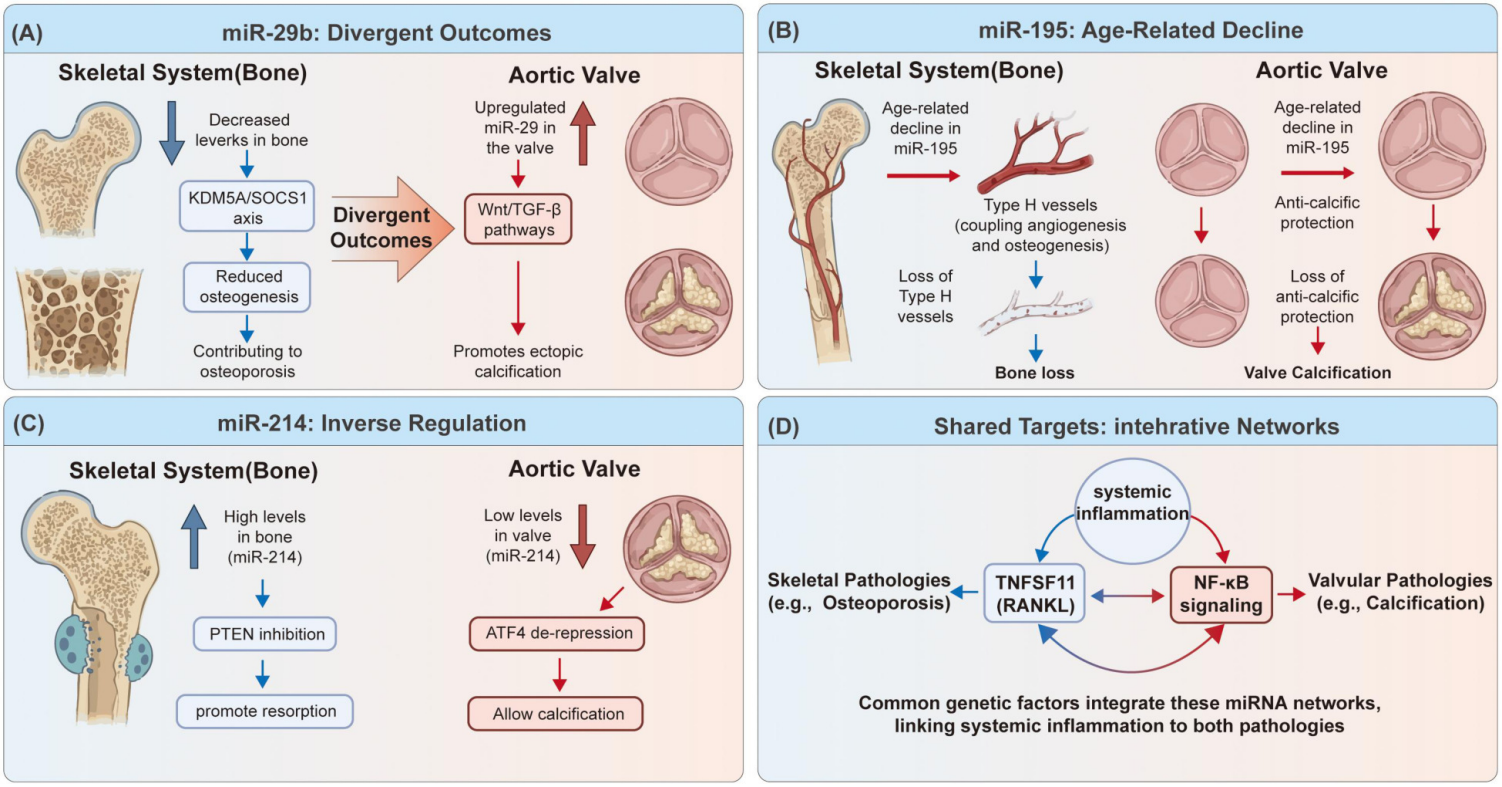
The miRNA-Mediated “Bone-Vascular Axis”: Shared Regulators with Divergent Outcomes. A schematic illustrating how key miRNAs differentially regulate homeostasis in the skeletal system (left) and the aortic valve (right). **(A)** miR-29b: Divergent Outcomes. Decreased levels of miR-29b in bone contribute to reduced osteogenesis and osteoporosis, while its upregulation in the valve promotes ectopic calcification via Wnt/TGF-β pathways. **(B)** miR-195: Age-Related Decline. Age-related decline in miR-195 leads to the loss of Type H vessels and subsequent bone loss, as well as the loss of anti-calcific protection in the valve, resulting in calcification. **(C)** miR-214: Inverse Regulation. High levels of miR-214 in bone promote resorption through PTEN inhibition, whereas low levels in the valve allow calcification via ATF4 de-repression. **(D)** Shared Targets: Integrative Networks. Common genetic factors and integrative networks involving systemic inflammation and NF-κB signaling link both skeletal and valvular pathologies.

Calcific aortic valve disease (CAVD) and osteoporosis (OP) frequently co-occur in the aging population, a clinical phenomenon often termed the bone-vascular axis (82, 83). While some epidemiological studies have confirmed that severe aortic calcification is an independent risk factor for osteoporosis (84), the molecular mechanisms linking these two distinct tissues remain complex. Recent bioinformatics analyses have identified shared genetic susceptibility loci, including TNFSF11 (RANKL), KYNU, and HLA-DMB, suggesting that immune-inflammatory dysregulation and metabolic pathways are common drivers for both pathologies (85). Within this shared landscape, microRNAs (miRNAs) have emerged as critical post-transcriptional switches that differentially regulate mineralization in valvular and skeletal tissues, often playing “dual roles” that lead to opposing clinical outcomes (Figure 1).

### 4.1 miR-29b: the “osteogenic” switch with opposing contexts

The miR-29 family functions as a master regulator of extracellular matrix synthesis and osteogenic differentiation. In the context of bone homeostasis, miR-29b is indispensable for osteoblast differentiation. Zhang et al. demonstrated that bone marrow-derived mesenchymal stem cell (BMSC)-derived extracellular vesicles (EVs) from osteoporotic patients contain significantly reduced levels of miR-29b-3p. Restoring miR-29b-3p promotes osteogenesis by targeting the epigenetic regulator KDM5A, thereby de-repressing the SOCS1-mediated inhibition of NF-κB signaling (86). Conversely, in the aortic valve, miR-29b is pathologically upregulated. It acts as a pro-calcific driver by repressing elastin and inhibitors of the Wnt/β-catenin pathway (e.g., TGF-β3), effectively “hijacking” the physiological bone-formation program to promote ectopic calcification (87, 88). Thus, a systemic imbalance of miR-29b—deficiency in the bone microenvironment and excess in the valve—contributes to the simultaneous manifestation of osteoporosis and CAVD.

### 4.2 miR-195 and the angiogenesis-osteogenesis coupling

The miR-497∼195 cluster represents a novel link between vascular biology and bone mass. In the skeletal system, Yang et al. identified that miR-195 is highly expressed in a specific subtype of capillary endothelium (Type H vessels) that couple angiogenesis with osteogenesis. The expression of miR-195 declines with age, leading to the loss of Type H vessels and subsequent age-related bone loss (osteoporosis) via the impairment of Notch and HIF-1α stability (89). In the aortic valve, miR-195 plays a protective, anti-calcific role by suppressing inflammatory and osteogenic signaling (e.g., VWF/p38 MAPK) (64). Therefore, the age-dependent systemic downregulation of miR-195 serves as a unified mechanism driving both vascular degeneration (loss of protection against calcification) and skeletal deterioration (uncoupling of osteogenesis).

### 4.3 miR-214: regulating the resorption-formation balance

miR-214 exemplifies the complex regulatory divergence in the bone-heart axis. In bone, miR-214 is a well-characterized inhibitor of bone formation and promoter of bone resorption. It suppresses osteoblast activity by targeting ATF4 and promotes osteoclastogenesis by targeting phosphatase and tensin homolog (PTEN) (90). Consequently, elevated miR-214 levels are associated with osteoporosis. In contrast, in the aortic valve, miR-214 functions as a “brake” on calcification; its expression is downregulated in calcified leaflets. Restoring miR-214 levels can inhibit valvular interstitial cell calcification by suppressing the same osteogenic target, ATF4 (42). This suggests that therapeutic strategies targeting miR-214 must carefully balance its opposing tissue-specific requirements.

### 4.4 miR-204: the Runx2 gatekeeper

miR-204 serves as a shared suppressor of Runx2, the master transcription factor for osteoblastogenesis. In the aortic valve, miR-204 is downregulated during disease progression, unleashing Runx2-dependent ectopic mineralization (45). In the skeletal system, miR-204 regulation is more nuanced; while it suppresses Runx2 to prevent aberrant osteogenesis, studies using puerarin (a phytoestrogen) have shown that inhibiting the TRPM3/miR-204 axis can actually promote osteoblast proliferation and differentiation in specific contexts, likely by modulating intracellular calcium flux (91). This highlights the potential of targeting upstream regulators of miRNAs, such as TRPM3, to modulate this axis.

### 4.5 Therapeutic implications

The identification of these shared miRNA networks and common gene targets (e.g., TNFSF11/RANKL) offers a theoretical basis for dual-purpose therapies. However, the complexity is highlighted by findings from Billig et al., where bisphosphonates (zoledronic acid) effectively reversed bone loss in an ovariectomized mouse model but failed to halt the progression of early-stage, wire-injury-induced aortic valve stenosis (92). This suggests that while the initiation mechanisms (e.g., inflammation, miRNA dysregulation) may be shared, the advanced propagation phases of CAVD might become independent of systemic bone turnover, necessitating stage-specific miRNA-based interventions (Table 1).

**Table 1:** Key microRNAs in calcific aortic valve disease (CAVD).

MicroRNA	Validated targets	Affected pathway/mechanism	Expression in CAVD tissue	Net effect	Cellular effects	Model	Reference
miR-101-3p	CDH11, SOX9	Wnt/β-catenin signaling	↑ (Up)	Pro-calcific	Promotes osteogenic differentiation	Human VICs	(23)
miR-638	Sp7 (Osterix)	Osteogenic signaling (Sp7-dependent)	↑ (Up)	Anti-calcific^a^	Accelerates VIC osteogenesis; inhibits autophagy	Human VICs	(35)
miR-214	ATF4, Sp7	Osteogenic signaling (ATF4-dependent)	↓ (Down)	Anti-calcific	Activates NLRP3 inflammasome; promotes inflammation	Human/Rat VICs	(42)
miR-204	Runx2, Osx	Osteogenic signaling (Runx2-dependent)	↓ (Down)	Anti-calcific	Increases MMP activity; promotes ECM degradation	Human/Mouse VICs	(45)
miR-449c-5p	Smad4	TGF-β/Smad signaling	↓ (Down)	Anti-calcific	Inhibits osteogenic differentiation (loss-of-function exacerbates calcification)	Human VICs	(52)
miR-138-5p	RUNX2	Wnt/β-catenin signaling	↓ (Down)	Anti-calcific	Suppresses VIC osteogenesis; protects against calcification	Human VICs	(56)
miR-195	VWF	p38 MAPK signaling	↓ (Down)	Anti-calcific	Inhibits osteogenic reprogramming of VICs	Human VICs	(64)
miR-22	CAB39	AMPK-mTOR signaling	↑ (Up)	Pro-calcific	Inhibits osteogenic differentiation	Human VICs	(27)
miR-181b	TIMP3	ECM Remodeling (MMP activation)	↑ (Up)^b^	Pro-calcific	Suppresses osteogenic differentiation	Human VECs	(76)
miR-29b	SOCS1	Inflammation (NF-κB/STAT3 signaling)	↑ (Up)	Pro-calcific	Inhibits inflammation and osteogenic differentiation	Mouse VICs	(68)

VICs, Valvular Interstitial Cells; VECs, Valvular Endothelial Cells; ECM, Extracellular Matrix; CDH11, Cadherin-11; SOX9, SRY-Box Transcription Factor 9; ATF4, Activating Transcription Factor 4; Runx2, Runt-related Transcription Factor 2; VWF, Von Willebrand Factor; TIMP3, Tissue Inhibitor of Metalloproteinases 3; SOCS1, Suppressor of Cytokine Signaling 1; MMP, Matrix Metalloproteinase.

a Note on miR-638: While highly expressed in calcified tissue, functional studies indicate it acts as an inhibitor of osteogenesis; its upregulation may represent a compensatory mechanism.

b Note on miR-181b: Upregulated specifically under conditions of disturbed flow/oscillatory shear stress in the fibrosa layer.

## 5 Discussion

CAVD is the most common valvular heart disease. The recognition of miRNAs as pivotal regulatory molecules in CAVD represents a paradigm shift in our understanding of this condition. MiRNAs add a crucial regulatory layer to these processes by modulating gene expression post-transcriptionally and orchestrating the interplay of multiple signaling pathways (77). Currently, the studies on CAVD mechanisms focusing on miRNAs has increased. Recent studies have shown that miRNAs are altered in the development of many disease (93, 94). And since 2015, more than 600 articles about miRNA-based therapeutics have been published (95). This suggests that they can be not only diagnostic, prognostic and predictive biomarkers, but also potential targets for new therapeutic agents (96). In other words, miRNA-focused strategies hold promise to fill the current gap in medical treatment for CAVD by enabling earlier diagnosis and offering molecular therapies to slow valvular degeneration.

Across the miRNAs identified in this review, a recurrent theme is their involvement in osteogenic signaling networks, particularly the BMP/Smad, TGF-β, and Wnt/β-catenin pathways. MiR-204, miR-449c-5p, and miR-138-5p exert anti-calcific effects by repressing osteogenic transcription factors such as Runx2, Osterix (Osx), and Sp7. These transcription factors are master regulators of osteoblast differentiation and are often hijacked in VICs under pro-calcific stimuli, leading to ectopic mineralization. Inhibition of these osteogenic cascades by specific miRNAs represents a protective mechanism against pathological calcification. Conversely, certain miRNAs act as pro-calcific drivers; for example, miR-101-3p and miR-22 promote the osteogenic trans-differentiation of VICs by downregulating endogenous inhibitors of osteogenic signaling and by suppressing autophagy and energy homeostasis. Through these mechanisms, pathological miRNAs tip the balance toward osteoblast-like activity in the valve, accelerating calcific nodule formation. This coordinated modulation of osteogenic pathways by various miRNAs highlights their central role in the calcification process of CAVD.

In addition to governing osteogenic reprogramming, several miRNAs influence inflammatory processes in the aortic valve—a key driver of early valvular pathology. Chronic inflammation is known to initiate and propagate CAVD by damaging the endothelium and promoting fibrotic and calcific changes. MiRNAs provide a mechanistic link between inflammatory signaling and these structural changes. For instance, miR-29b has been shown to promote activation of the NLRP3 inflammasome, an innate immune complex implicated in sterile inflammation and biomineralization of the valve. By targeting negative regulators of the inflammasome (such as SOCS1, as identified in prior studies), miR-29b can tip the immune response towards a pro-inflammatory, pro-calcific state in the valve. Another example is miR-181b, which connects mechanical stimuli to inflammatory remodeling: under conditions of disturbed flow (a hallmark of valvular endothelial stress), miR-181b is upregulated and in turn downregulates TIMP3, the tissue inhibitor of metalloproteinases. The loss of TIMP3 leads to unrestrained MMP activity, contributing to extracellular matrix remodeling and fibrotic thickening of the valve. Through these actions, miR-181b effectively links hemodynamic stress to an inflammatory-fibrotic response. These findings underscore how miRNAs act as molecular bridges between mechanical forces, inflammatory signaling, and the cellular behaviors that cause structural valve degeneration. In summary, miRNAs integrate multiple pathological stimuli—from osteogenic cues to inflammatory and mechanical factors—to orchestrate the complex cellular reprogramming that underlies CAVD progression. However, it is important to acknowledge that a significant proportion of these mechanistic insights relies on rodent model, which do not fully recapitulate the complex hemodynamics, layer composition, and slow progression of human CAVD (97). Therefore, caution is warranted when extrapolating these species-specific miRNA profiles to human pathology.

Despite the promise of miRNA-based diagnostics and therapeutics, transitioning from bench to bedside faces several translational challenges. First, technical variability and inconsistencies across studies complicate clinical adoption. As highlighted by systematic reviews, the directionality of miRNA expression (e.g., miR-214) can vary depending on the specific disease stage or osteogenic induction conditions (77), and the lack of standardized protocols for sample handling (e.g., serum vs. plasma) introduces significant bias (98). Second, the pleiotropic nature of miRNAs creates a risk of unpredictable downstream consequences, particularly given the “bone-vascular axis.” For instance, while Zeng et al. demonstrated that inhibiting the TRPM3/miR-204 axis with Puerarin promotes osteoblast differentiation and reverses bone loss (91), systemic inhibition of miR-204 could theoretically exacerbate aortic valve calcification, where miR-204 functions as a critical repressor of Runx2. Similarly, indiscriminate inhibition of miR-214 to treat osteoporosis might inadvertently unleash ATF4-mediated calcification in the valve (90). Third, delivery remains a major hurdle due to the avascular nature of the valve and rapid hemodynamic washout. To overcome stability issues (e.g., RNase degradation) and improve tissue uptake, advanced delivery vectors are required. Lipid nanoparticles (LNPs) and polymer-based systems [e.g., poly (lactic-co-glycolic acid) (PLGA), Chitosan] have shown promise in cancer trials for delivering siRNA/miRNA by enhancing cellular penetration and endosomal escape (7, 77). engineered extracellular vesicles (EVs), which naturally cross biological barriers and exhibit low immunogenicity, represent a novel strategy for targeted delivery or as source of specific biomarkers, as demonstrated by the isolation of brain-cell-derived EVs from plasm (78). Furthermore, defining the therapeutic window is critical. As evidenced by the failure of bisphosphonates in the SALTIRE II trial and recent murine models by Billig et al., interventions likely need to target the initiation phase—governed by inflammation and early remodeling—rather than the irreversible calcific stage (92). In this context, multi-target agents identified through bioinformatics, such as 17-beta-estradiol (targeting KYNU) or Vitamin D3, might offer early dual-protection for both bone and heart (85). Finally, applying these molecular insights to bioprosthetic valves—for instance, by coating leaflets with miRNA-loaded nanoparticles—could suppress immune rejection and extend device durability (8).

In conclusion, miRNAs have emerged as critical modulators of the cellular pathways driving CAVD, controlling processes from osteogenic differentiation to inflammation. While the complex mechanisms of their action in valve pathology are not yet fully elucidated, studies on microRNAs in CAVD are still limited, making it difficult to construct a unifying conceptual framework or integrated signaling network. Continued research is warranted to map these pathways in greater detail. Such efforts will deepen our understanding of CAVD pathogenesis and could unveil novel therapeutic targets to combat this disease. Ultimately, translating miRNA insights into the clinic—through biomarker development or miRNA-based therapies—holds considerable promise for improving the management of CAVD, a disease for which new medical interventions are urgently needed.

## 6 Conclusion

MiRNAs play an indispensable role in the initiation and progression of CAVD by modulating endothelial function, inflammation, ECM remodeling, and VICs osteogenic differentiation. A growing body of experimental evidence highlights specific miRNAs as central nodes in the molecular networks governing valve calcification. While significant strides have been made in understanding their mechanistic roles, translating these findings into clinical practice remains a key challenge. Future research should focus on validating miRNA biomarkers in large patient cohorts, elucidating cell- and tissue-specific functions, and developing targeted delivery systems for therapeutic modulation. Importantly, the shared regulatory pathways between CAVD and skeletal disorders such as osteoporosis open new opportunities for dual-purpose interventions. As we continue to unravel the complex miRNA-mediated regulatory landscape, these small RNAs hold great promise as both diagnostic and therapeutic tools in the fight against calcific aortic valve disease.
